# Effects of post-interventional antiplatelet therapy on angiographic vasospasm, delayed cerebral ischemia, and clinical outcome after aneurysmal subarachnoid hemorrhage: a single-center experience

**DOI:** 10.1007/s10143-021-01477-6

**Published:** 2021-01-25

**Authors:** Claudia Ditz, Björn Machner, Hannes Schacht, Alexander Neumann, Peter Schramm, Volker M. Tronnier, Jan Küchler

**Affiliations:** 1 Department of Neurosurgery, Universitätsklinikum Schleswig-Holstein, Campus Lübeck, Ratzeburger Allee 160, 23538 Lübeck, Germany; 2 Department of Neurology, Universitätsklinikum Schleswig-Holstein, Campus Lübeck, Lübeck, Germany; 3 Department of Neuroradiology, Universitätsklinikum Schleswig-Holstein, Campus Lübeck, Lübeck, Germany

**Keywords:** Antiplatelet therapy, Cerebral vasospasm, Delayed cerebral ischemia, Endovascular treatment, Aneurysmal subarachnoid hemorrhage

## Abstract

Platelet activation has been postulated to be involved in the pathogenesis of delayed cerebral ischemia (DCI) and cerebral vasospasm (CVS) after aneurysmal subarachnoid hemorrhage (aSAH). The aim of this study was to investigate potentially beneficial effects of antiplatelet therapy (APT) on angiographic CVS, DCI-related infarction and functional outcome in endovascularly treated aSAH patients. Retrospective single-center analysis of aSAH patients treated by endovascular aneurysm obliteration. Based on the post-interventional medical regime, patients were assigned to either an APT group or a control group not receiving APT. A subgroup analysis separately investigated those APT patients with aspirin monotherapy (MAPT) and those receiving dual treatment (aspirin plus clopidogrel, DAPT). Clinical and radiological characteristics were compared between groups. Possible predictors for angiographic CVS, DCI-related infarction, and an unfavorable functional outcome (modified Rankin scale ≥ 3) were analyzed. Of 160 patients, 85 (53%) had received APT (*n* = 29 MAPT, *n* = 56 DAPT). APT was independently associated with a lower incidence of an unfavorable functional outcome (OR 0.40 [0.19–0.87], *P* = 0.021) after 3 months. APT did not reduce the incidence of angiographic CVS or DCI-related infarction. The pattern of angiographic CVS or DCI-related infarction as well as the rate of intracranial hemorrhage did not differ between groups. However, the lesion volume of DCI-related infarctions was significantly reduced in the DAPT subgroup (*P* = 0.011). Post-interventional APT in endovascularly treated aSAH patients is associated with better functional outcome at 3 months. The beneficial effect of APT might be mediated by reduction of the size of DCI-related infarctions.

## Introduction

Delayed cerebral ischemia (DCI) is a severe complication after aneurysmal subarachnoid hemorrhage (aSAH) and represents the most important predictor of mortality and morbidity in patients who survive the acute bleeding event [18, 37]. DCI usually occurs between 4 and 10 days after the initial hemorrhage. It is clinically defined by the development of new neurological deficits or a sudden decrease of consciousness [50] and may lead to secondary cerebral infarction and permanent disability [35, 47, 50]. The pathophysiological mechanisms involved in DCI are complex and still not completely understood. Cerebral vasospasm (CVS), characterized by angiographic vasoconstriction of arterial blood vessels, plays an important role in the development of DCI [10, 17]. However, current research proposes a multifactorial nature of DCI beyond the traditionally emphasized CVS. Several mechanisms have been postulated to be involved including cortical spreading depolarization, inflammatory response, activation of the coagulation cascade, and microthrombosis [3, 28, 38, 49]. In particular, increased platelet activation after SAH is considered to contribute to the development of DCI. Endothelial damage from aneurysm rupture leads to immediate platelet activation and aggregation with consecutive release of cytokines and vasoactive substances from within the platelet granules. These processes do not only cause vasoconstriction and the formation of microthrombi but also contribute to the activation of inflammation [48]. Accordingly, an increased platelet activity after aSAH with consecutive release of thromboxane A2 was found to be associated with DCI and the occurrence of angiographic CVS [25, 26, 31]. Moreover, the antiplatelet activity of the endothelium is reduced after aSAH resulting in an increased platelet adhesion and formation of microthrombi [30]. Previous studies suggest that microthrombi/-emboli play an essential role in the development of DCI-related infarction [34, 41, 42, 48].

Several clinical studies have therefore evaluated possible preventive effects of different antiplatelet agents such as aspirin on the development of CVS, DCI, and functional outcome after aSAH [15, 20, 24, 45, 46]. However, the results of these studies are contradictory and quite heterogeneous with regard to the investigated antiplatelets, the start and duration of treatment, and the investigated endpoints. Moreover, most of the older studies in the pre-ISAT era primary report on surgically treated patients, whereas endovascularly treated patients are underrepresented. Accordingly, appropriate treatment recommendations regarding the use of antiplatelets in aSAH patients are still missing. However, in view of the current developments toward an increasing role of endovascular aneurysm treatments, appropriate risk-benefit assessments and recommendations regarding APT in patients with acute SAH are needed. A growing number of aSAH patients require post-interventional APT with aspirin or even dual antiplatelet therapy (DAPT, aspirin plus clopidogrel), due to stent or flow diverter–assisted aneurysm treatments. Hence, recent series focused on the effects of post-interventional APT among endovascularly treated aSAH patients [4, 11, 29, 51].

The purpose of this study was to retrospectively evaluate the effects of APT (aspirin with/without additional clopidogrel) on functional outcome, the occurrence of angiographic CVS, and the prevention of DCI-related infarction in a collective of endovascularly treated aSAH patients. In particular, we compared characteristic patterns of angiographic CVS and DCI-related infarction between the groups and assessed the risk of hemorrhagic complications. We aimed to provide additional guidance to the ongoing debate on the value of APT in aSAH patients.

## Patients and methods

This study was approved by the Institutional Review Board (20-257). For this type of retrospective study, informed consent is not required. We screened all consecutive patients who were admitted to our institution between January 2011 and December 2019 for the following inclusion criteria: (i) SAH secondary to the rupture of an intracranial aneurysm, (ii) endovascular aneurysm treatment. The exclusion criteria were as follows: surgical treatment of the aneurysm, no treatment of the aneurysm due to devastating aSAH, early death (< 7 days after admission), aSAH associated with arteriovenous malformation or mycotic aneurysm, coiling of the aneurysm with the need for secondary stenting later within the acute clinical course, stent-assisted coiling without subsequent administration of antiplatelets, evidence of CVS on admission angiography, and the use of heparin, e.g., due to deep-vein thrombosis or acute coronary syndrome.

General demographic information and clinical and radiological data were collected by chart review. Patients receiving post-interventional antiplatelet medication (aspirin ± clopidogrel) were assigned to the “APT group,” while the “control group” consisted of patients without antiplatelet medication after endovascular aneurysm repair. A subgroup analysis was performed within the “APT” group investigating patients with either mono- (aspirin) or dual (aspirin plus clopidogrel) APT.

Hemorrhagic intracranial complications were assessed on regular follow-up non-enhanced computed tomography (NCT) and categorized into minor and major bleeding events, whereas major events were defined as new intracranial hemorrhage that led to a clinical deterioration and/or necessitated additional surgery.

Functional outcome was assessed at 3 months after discharge using the modified Ranking Scale (mRS) and dichotomized into favorable (mRS ≤ 2) and unfavorable outcome (mRS ≥ 3).

### Definition and analysis of DCI, DCI-related infarction, and angiographic CVS

DCI was defined clinically as the development of new focal neurological deficits and/or a decrease in Glasgow Come Scale ≥ 2 points, lasting for more than 1 h (delayed ischemic neurologic deficit, DIND), and/or by imaging criteria as new DCI-related infarction on follow-up NCT or magnetic resonance imaging (MRI) [50]. In analgosedated patients, who evade neurological assessments, the definition of DCI was expanded to include events of functional deterioration such as a refractory decrease in PBrO_2_ below 15 mmHg or DCI-related hypoperfusion on PCT as defined below. Events of clinical or functional DCI were documented and compared between the groups. However, as our study cohort consisted of awake and unconscious/analgosedated patients, data on clinical or functional DCI were only partially available and difficult to compare. Thus, only the occurrence of DCI-related infarction, as defined by radiological criteria, was analyzed as an independent endpoint in multivariable analysis as this outcome parameter was assessable in all patients and has the greatest impact on clinical outcome.

DCI-related infarction was defined as new hypodense lesions on follow-up CT or as new diffusion-restricted areas on MRI not attributable to other causes. Pre-existing infarctions and infarctions related to the primary brain damage, cerebral herniation, or the initial aneurysm treatment were excluded. Hypodensities resulting from intracerebral hematoma or surgical interventions were not regarded as infarction.

DCI-related infarctions were grouped into the following categories: single or multiple vascular territories, cortical or deep location, and unilateral or bilateral occurrence [33].

The NCT scans of all patients were inspected by two experienced neuroradiologists, and DCI-related infarctions were manually delineated on the individual patient image using the MRIcron software (http://www.mricro.com/mricron) [36]. Next, the delineated lesion of every patient was normalized into the standardized MNI (Montreal Neurological Institute) space using the clinical toolbox in SPM (Statistical Parametric Mapping, version 12, http://www.fil.ion.ucl.ac.uk/spm; Wellcome Trust Centre for Neuroimaging, London, UK), implemented in Matlab® 2018B (MathWorks®, Natick, MA). Using MRIcron, lesion overlap images were created for each group to illustrate the location, distribution, and extent of DCI-related infarctions. Furthermore, the individual lesion volume was derived and used for further statistical analyses.

Angiographic CVS was defined as the occurrence of new arterial vessel narrowing on follow-up digital subtraction angiography (DSA) (compared with the vessel diameter in the initial DSA performed on admission). Severity and distribution of angiographic CVS were assessed for each vessel by the neuroradiologist who performed the intervention and verified retrospectively by a second experienced interventional neuroradiologist. The number of affected vessels per patient and the affected vessel segments (proximal/distal) in each DSA as well as the number and location of intra-arterial nimodipine (IAN) treatments and/or balloon angioplasty were collected. Proximal angiographic CVS was defined as CVS in the internal carotid artery, the vertebral or basilar artery, and within the first segments of the anterior, middle, and posterior cerebral artery. The degree of angiographic CVS was rated as “mild” when narrowing of the arterial diameter < 29%, “moderate” when 30 to 49%, and “severe” when ≥ 50%, each compared with the vessel diameter on the initial angiography.

### Clinical management of aSAH patients

All patients with aSAH were managed in the neurointensive care unit under the care of the neurosurgery service and in accordance with current recommendations [9]. Four-vessel DSA was performed in all patients in order to evaluate the presence of a ruptured aneurysm. In case of acute hydrocephalus or severe IVH, cerebrospinal fluid (CSF) drain was placed prior to the endovascular intervention. The choice of endovascular treatment was made individually upon interdisciplinary assessment based on the aneurysm location and configuration as well as on demographic and clinical factors. Endovascular aneurysm treatment was performed within 48 h after admission.

The decision about the use of APT after coil embolization was made by the treating neuroradiologist depending on the expected risk of thromboembolic complications (e.g., wide aneurysm base, protrusion of a coil in the vessel lumen). If indicated after coil embolization, aspirin was administrated in a daily dose of 100 mg for approximately 4 to 12 weeks. If remodeling stents or flow diverters were used for acute treatment, intravenous tirofiban infusion was started with a basal-bolus regimen of 0.4 μg/kg/min for 30 min followed by a 0.10 μg/kg/min maintenance infusion until post-interventional complications were ruled out by follow-up NCT scans. If necessary, tirofiban infusion was paused to allow the placement of an external ventricular drain (EVD) or invasive neuromonitoring probes. Subsequently, loading with 300 mg aspirin and 450 mg clopidogrel was performed and followed by a daily maintenance dose of 100 mg aspirin and 75 mg clopidogrel. DAPT was continued for at least 6 weeks while aspirin use was continued in most cases. Platelet function tests were not routinely performed in all included patients.

After aneurysm treatment, the patients were kept normovolemic with a mean arterial pressure (MAP) above 80 mmHg. Standard antithrombotic therapy included subcutaneous low-dose heparin starting from day 1 after aneurysm treatment. All patients received nimodipine, administered orally at a dose of 60 mg every 4 h or intravenously with 1–2 mg of nimodipine per hour.

All patients were monitored for DCI and CVS with serial neurologic examinations and daily transcranial Doppler (TCD) measurements of flow velocities (FVs) in the large cerebral arteries.

Routine monitoring for DCI and CVS included serial neurologic examinations in awake patients and daily transcranial Doppler (TCD) measurements. Analgosedated patients such as patients with poor-grade sSAH (World Federation of Neurosurgical Societies (WFNS) grade 4–5) received an invasive multimodal neuromonitoring for intracranial pressure (ICP), cerebral perfusion pressure (CPP), and brain tissue oxygenation (PBrO_2_). The neuromonitoring probes were placed in the frontal watershed area of the hemisphere ipsilateral to the ruptured aneurysm and in most cases additionally in the contralateral hemisphere. An increase of TCD mean FV of 150 cm/s or an increase of FV by 50 cm/s over 24 h, as well as a refractory decrease in PBrO_2_ below 15 mmHg, was considered as indications of CVS or DCI.

Follow-up imaging included an early post-interventional NCT (within 24 h after aneurysm treatment) in all patients. Further follow-up NCT scans were performed during the hospital stay depending on the clinical indication. Additional perfusion CT (PCT) examinations were performed in the event of clinical DCI (awake patients) or in case of functional suspicion of DCI or CVS. In analgosedated patients, PCTs were also performed routinely every 3 to 4 days according to our internal protocol [12]. Perfusion maps were qualitatively/semi-quantitatively evaluated via visual inspection by two board-certified neuroradiologists to detect new DCI-related perfusion deficits, defined as areas of prolonged TTD not related to other causes. The TTD parameter map was chosen since it was formerly described to provide the best image quality and the highest contrast for delineating ischemic areas [13].

In case of clinical or functional DCI, hyperdynamic therapy was initiated as recommended [9] by raising the systolic arterial blood pressure above 180 mmHg by the use of crystalloids and/or vasopressors. If the conservative treatment failed to improve the patients’ clinical or functional condition, DSA was performed to confirm angiographic CVS and to initiate ERT upon interdisciplinary decision.

### Statistical analysis

Data analysis was performed using the software IBM® SPSS® 25 (IBM; Armonk, NY, USA). Continuous data are presented as medians and interquartile ranges (IQRs) while categorical data are presented by counts and percentages. Categorical variables were analyzed using the chi-square test, or, if applicable, with the Fisher exact test. Continuous variables were analyzed using the Mann–Whitney *U* test for non-normally distributed data. A *P* value ≤ 0.05 was considered statistically significant.

We used a multivariable logistic regression with backward stepwise selection to analyze factors that might be associated with the occurrence of angiographic CVS, DCI-related infarction, and functional outcome. All factors that had *P* values < 0.05 in a previous univariable testing were included. Variables with *P* values <0.05 were accepted as independent predictors. Odds ratios and associated 95% confidence intervals were reported for both the univariable and multivariable analyses.

## Results

Between January 2011 and December 2019, a total of 307 patients with aSAH were treated at our institution and 282 patients received aneurysm obliteration. Overall, 93 patients were treated surgically (33%), while 189 patients received endovascular aneurysm treatment (67%). Middle cerebral artery aneurysms (*n* = 70) were predominantly secured by surgical clipping (*n* = 46, 66%). After excluding 29 patients due to the above-mentioned exclusion criteria, 160 patients with aSAH and endovascular aneurysm treatment were included in the final analysis. Eighty-five patients (53%) received APT (APT group), 1/3 of them had aspirin monotherapy (*n* = 29/85) and 2/3 a DAPT consisting of aspirin and clopidogrel (*n* = 56/85). The remaining 75 patients (47%) received no APT and constituted the control group.

Post-interventional platelet function test was performed in 35/85 APT patients (41%), all of whom received DAPT due to stent-assisted coiling. The majority of patients (29/35, 83%) showed a sufficient treatment response to both aspirin and clopidogrel, while 6 patients (17%) responded insufficiently to APT (non-responsiveness to (i) both aspirin and clopidogrel *n* = 3, (ii) aspirin only *n* = 1, (iii) clopidogrel only *n* = 2). At our institution, APT responder status was not assessed in case of post-interventional aspirin monotherapy.

The clinical characteristics of either group are shown in Table 1. There were no significant differences between the groups with regard to age, sex, WFNS and Fisher grade, acute hydrocephalus, aneurysm localization, or laterality. CVS drainage was required in 76% of the control group and in 64% of the APT group. EVD was placed in the majority of cases in both groups (74% in the control group vs. 63% in the APT group), and most patients received the CVS drain prior to the endovascular aneurysm treatment (65% in the control group vs. 59% in the APT group).

**Table 1:** Clinical characteristics of the study cohort

Parameter	Control group, *n* = 75	APT group, *n* = 85	*P* value
Age in years, median (IQR)	55 (47–65)	54 (48–65)	0.801
Female sex, *n* (%)	51 (68)	64 (75)	0.379
*WFNS grade, n (%)*			
I–III	46 (61)	60 (71)	0.243
IV–V	29 (39)	25 (29)	
*Fisher scale, n (%)*			
0–2	9 (12)	17 (20)	0.201
3–4	66 (88)	68 (80)	
Intraventricular hemorrhage, *n* (%)	31 (41)	34 (40)	0.496
Intracerebral hemorrhage, *n* (%)	14 (19)	9 (11)	0.178
Acute hydrocephalus requiring CSF drain, *n* (%)	57 (76)	54 (64)	0.171
*Aneurysm location, n (%)*			
ICA	11 (15)	17 (20)	0.209
PComA	9 (12)	3 (4)	
AComA and ACA	30 (40)	29 (34)	
MCA	7 (9)	12 (14)	
Posterior circulation	18 (24)	24 (28)	
*Aneurysm laterality, n (%)*^a^			
Right side	22 (29)	23 (27)	0.406
Left side	19 (25)	30 (35)	
Endovascular aneurysm treatment modality, *n* (%)			
Coil embolization	75 (100)	29 (34)	*< 0.001*
WEB Device	0 (0)	2 (2)	
Stent-assisted coil embolization	0 (0)	51 (60)	
Flow diverter	0 (0)	3 (4)	
*Peri-interventional complications, n (%)*	10 (13)	12 (14)	0.536
Thromboembolic infarction	4/10 (40)	5/12 (42)	
Bleeding complication	6/10 (60)	3/12 (25)	
Vascular dissection	0/10 (0)	1/12 (8)	
Intraprocedural coil dislocation	0/10 (0)	3/12 (25)	
Decompressive craniectomy, *n* (%)	7 (9)	4 (5)	0.350
VP shunt, *n* (%)	16 (21)	20 (24)	0.850
Length of stay (days), median (IQR)	20 (16–24)	20 (17–25)	0.853
In-hospital mortality, *n* (%)	8 (11)	5 (6)	0.386
*Intracranial bleeding events, n (%)*	5 (7)	5 (6)	0.114
Minor	5 (7)	2 (2)	
Major	0 (0)	3 (4)	
*Clinical or functional signs of DCI or CVS, n (%)*			
Significant increase of FV in TCD	25 (33)	30 (35)	0.868
DIND in awake patients	17/36 (47)	22/54 (41)	0.665
Functional deterioration			
PBrO_2_ < 15 mmHg in analgosedated patients	19/34 (56)	10/27 (37)	0.198
DCI-related hypoperfusion in PCT	34/53 (64)	18/53 (34)	*0.003*
*DCI-related infarction, n (%)*	20 (27)	21 (25)	0.857
Vascular territories of DCI-related infarction, *n* (%)			
Single vascular territory	10/20 (50)	10/21 (48)	0.563
Multiple vascular territories	10/20 (50)	11/21 (52)	
Location of DCI-related infarction, *n* (%)			
Cortical	14/20 (70)	12/21 (57)	0.493
Deep	0/20 (0)	1/21 (5)	
Combined	6/20 (30)	8/21 (38)	
Laterality of DCI-related infarction, *n* (%)			
Unilateral	13/20 (65)	14/21 (66)	0.585
Right side	10/20	8/21	
Left side	3/20	6/21	
Bilateral	7/20 (35)	7/21 (33)	
Lesion volume (cm^3^), median (IQR)	78.5 (24.7-282.6)	33.7 (6.1–107.5)	0.090

Statistically significant differences are made italics (*P* < 0.05)

*ATP* antiplatelet therapy, *IQR* interquartile range, *WFNS* World Federation of Neurosurgical Societies, *CSF* cerebrospinal fluid, *ICA* internal carotid artery, *PComA* posterior communicating artery, *AComA* anterior communicating artery, *ACA* anterior cerebral artery, *MCA* middle cerebral artery, *WEB* Woven EndoBridge; *VP* ventriculoperitoneal, *DCI* delayed cerebral ischemia, *CVS* cerebral vasospasm, *FV* flow velocity, *TCD* transcranial Doppler, *DIND* delayed ischemic neurologic deficit, *PBrO*_*2*_ brain tissue oxygenation, PCT perfusion computed tomography

^a^Basilar artery and AComA aneurysms were excluded

As expected, there was a statistically significant difference with regard to the modality of endovascular aneurysm treatment (*P* < 0.001) between the groups.

Treatment-related complications occurred in a similar percentage of patients in either group (13% in the control group vs. 14% in the APT group), most of which were thromboembolic infarction and peri-interventional bleeding complications.

Clinical complications such as decompressive craniectomy or the need for ventricular peritoneal (VP) shunting as well as the in-hospital mortality (11% in the control group vs. 6% in the APT group, *P* = 0.386) were similar between the groups. There was no significant difference regarding the incidence of any intracranial bleeding event (7% in the control group vs. 6% in the APT group, *P* = 0.114). However, while minor bleeding events occurred more often in the control group (7% vs. 2%), major bleeding events were only observed in APT patients (*n* = 3, 4%). One patient developed space-occupying epi- and subdural hematoma after decompressive craniectomy, while two patients developed large intracerebral hematoma after EVD or VP shunt placement, respectively. One patient died during hospitalization, and two patients had unfavorable outcome after 3 months.

Eighty-six patients (54%) were awake and neurologically assessable during the clinical course. In these patients, DIND occurred in 39 cases (control group 17/36, 47%; APT group 22/54, 41%; *P* = 0.665). On the other hand, 74 patients (46%) of the study cohort (control group *n* = 42, 56%; APT group *n* = 32, 38%) were (temporary) analgosedated and mechanically ventilated. Sixty-one patients (38%) (control group *n* = 34, 45%; APT group *n* = 27, 32%) required long-term analgosedation due to poor-grade aSAH (WFNS IV-V) or complications in the acute clinical course and received invasive neuromonitoring including PBrO_2_ which was placed bilaterally in the majority of patients (44/61, 72%). Twenty-nine patients showed functional deterioration with a decrease of PBrO_2_ (control group 19/34, 56%; APT group 10/27, 37%; *P* = 0.198). PCT examinations were performed in 106 patients (control group 53/75, 71%; APT group 53/85, 62%). DCI-related hypoperfusion was observed significantly more often in the control group (34/53, 64%) compared with the APT group (18/53, 34%; *P* = 0.003). Routine TCD measurements showed a significant increase in FV in about 1/3 of the patients (control group *n* = 25, 33%; APT group *n* = 30, 35%; *P* = 0.868).

Angiographic CVS was present in 41% of patients in either group (*P* = 1.000; Table 2). The further analysis of characteristic angiographic CVS pattern showed a similar distribution of the investigated parameters between the control and APT group. In particular, there were no differences between the groups with regard to the number of affected vessels and the severity of angiographic CVS. Moreover, a similar number of patients received ERT, mostly IAN applications (Table 2). Of the 51 patients treated with stent-assisted coiling, 19 patients developed symptomatic angiographic CVS (37%). In the majority of cases (*n* = 15/19, 79%), the stented vessel segment was not or only mildly affected by CVS. In case of unilateral occurrence, CVS was primary detected ipsilateral to the ruptured aneurysm (*n* = 24/27, 88%).

**Table 2:** Parameters regarding angiographic CVS patterns in the study cohort

Parameter	Control group (*n* = 75), *n* (%)	APT group (*n* = 85), *n* (%)	*P* value
Occurrence of angiographic CVS	31 (41)	35 (41)	1.000
No. of affected vessels per patient			
1 vessel	2/31 (6)	4/35 (11)	0.709
2–3 vessels	12/31 (39)	13/35 (37)	
> 3 vessels	17/31 (55)	16/35 (46)	
Affected vessel segment per patient			
Proximal	30/31 (98)	30/35 (86)	0.784
Distal	23/31 (74)	23/35 (66)	
Lateralization of CVS			
Unilateral	17/31 (55)	21/35 (60)	0.804
Bilateral	14/31 (45)	14/35 (40)	
Localization of CVS			
Anterior circulation	31/31 (100)	34/35 (97)	0.403
Posterior circulation	10/31 (31)	8/35 (23)	
Severity of CVS			
Mild to moderate	8/31 (26)	13/35 (37)	0.429
Severe	23/31 (74)	22/35 (63)	
Endovascular rescue therapy	31 (41)	35 (41)	
IAN	30/31 (98)	32/35 (91)	0.615
TBA	12/31 (39)	9/35 (26)	0.426

*APT* antiplatelet therapy, *CVS* cerebral vasospasm, *IAN* intra-arterial nimodipine treatment, *TBA* transarterial balloon angioplasty

In the multivariable analysis of factors associated with the occurrence of angiographic CVS (Table 3), poor WFNS grade independently increased the risk for angiographic CVS in our cohort (*P* = 0.026, OR = 2.15, 95% CI 1.10–4.23), while male sex showed a non-significant statistical trend toward a reduced risk for angiographic CVS (*P* = 0.055, OR = 0.48, 95% CI 0.23–1.02). The use of APT had no significant impact on the occurrence of angiographic CVS.

**Table 3:** Logistic regression analysis of factors associated with the occurrence of angiographic CVS or DCI-related infarction

	Angiographic CVS				DCI-related infarction			
Parameter	Univariable analysis		Multivariable analysis		Univariable analysis		Multivariable analysis	
	OR [CI 0.95]	*P* value	OR [CI 0.95]	*P* value	OR [CI 0.95]	*P* value	OR [CI 0.95]	*P* value
Older age (> 55 years)	0.85 [0.45–1.59]	0.610	–	–	1.88 [0.91–3.88]	0.087	–	–
Male sex	*0.48 [0.23–1.00]*	*0.049*	0.48 [0.23–1.02]	0.055	0.54 [0.23–1.28]	0.159	–	–
WFNS grade 4–5	*2.16 [1.11–4.22]*	*0.023*	*2.15 [1.10–4.23]*	*0.026*	*3.63 [1.73–7.6]*	*< 0.001*	*3.63 [1.73–7.61]*	*0.001*
Fisher scale 3	0.77 [0.41–1.46]	0.426	–	–	0.87 [0.43–1.78]	0.706	–	–
Occurrence of IVH	1.41 [0.74–2.67]	0.298	–	–	*2.69 [1.30–5.56]*	*0.008*	1.56 [0.65–3.73]	0.317
Aneurysm localization Posterior circulation	0.55 [0.26–1.16]	0.117	–	–	0.40 [0.15–1.02]	0.056	–	–
Antiplatelet therapy	0.99 [0.53–1.87]	0.984	–	–	0.90 [0.44–1.84]	0.777	–	–

Data comparisons were made using univariable and multivariable analysis. Statistically significant differences are made italics (*P* < 0.05)

*CVS* cerebral vasospasm, *DCI* delayed cerebral ischemia, *OR* Odds ratio, *CI* confidence interval, *WFNS* World Federation of Neurosurgical Societies, *IVH* intraventricular hemorrhage

**Fig. 1:**
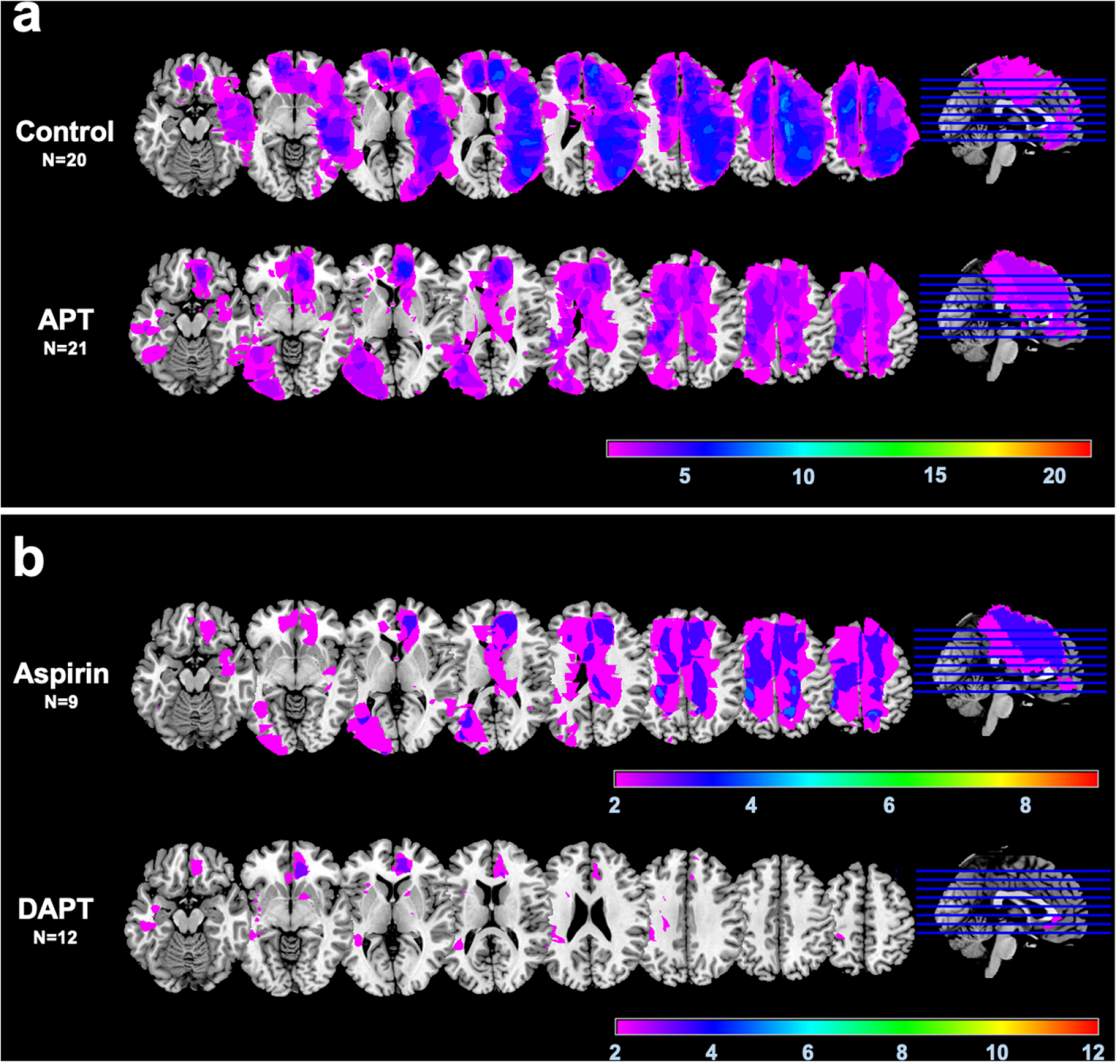
Lesion overlay plots showing DCI-related cerebral infarctions in the control and the APT group (**a**) and, in a further subgroup analysis, infarct lesions inpatients receiving aspirin monotherapy or dual APT (aspirin plus clopidogrel) (**b**). The color bar indicates the number of overlapping lesions (minimum *n* = 2)

Regarding the incidence of DCI-related infarctions in our collective, we found no significant differences between the study groups (27% in the control group vs. 25% in the APT group, *P* = 0.875; Table 1). A detailed analysis of the infarct characteristics showed comparable patterns with respect to the number of vascular territories involved, the location or the laterality (Table 1). Although the median lesion volume did not differ significantly between the groups, we found a statistical trend toward higher absolute values in the control group 78.5 cm^3^ (IQR 24.7–282.6) vs 33.7 cm^3^ (IQR 6.1–107.5) in the APT group (*P* = 0.090). The lesion overlay plots of patients with DCI-related infarctions are shown in Fig. 1. It visually appeared that the patients in the control group developed more often larger areas of infarction, especially in the left hemisphere (Fig. 1a). In the lesion overlap image of the two subgroups of APT patients (aspirin mono APT vs. DAPT), it visually appeared that patients with DAPT had especially small lesion overlaps (Fig. 1b). This impression could also be statistically supported, because the median lesion volume was significantly smaller in the DAPT subgroup (14.3 cm^3^ (IQR 5.1–57.8) than in the control group (78.5 cm^3^ (IQR 24.7–282.6), *P* = 0.011).

Multivariable analyses (Table 3) showed that a poor WFNS grade at admission was the only independent predictor of DCI-related infarction in our cohort (*P* = 0.001, OR = 3.63, 95% CI 1.73–7.61).

**Fig. 2:**
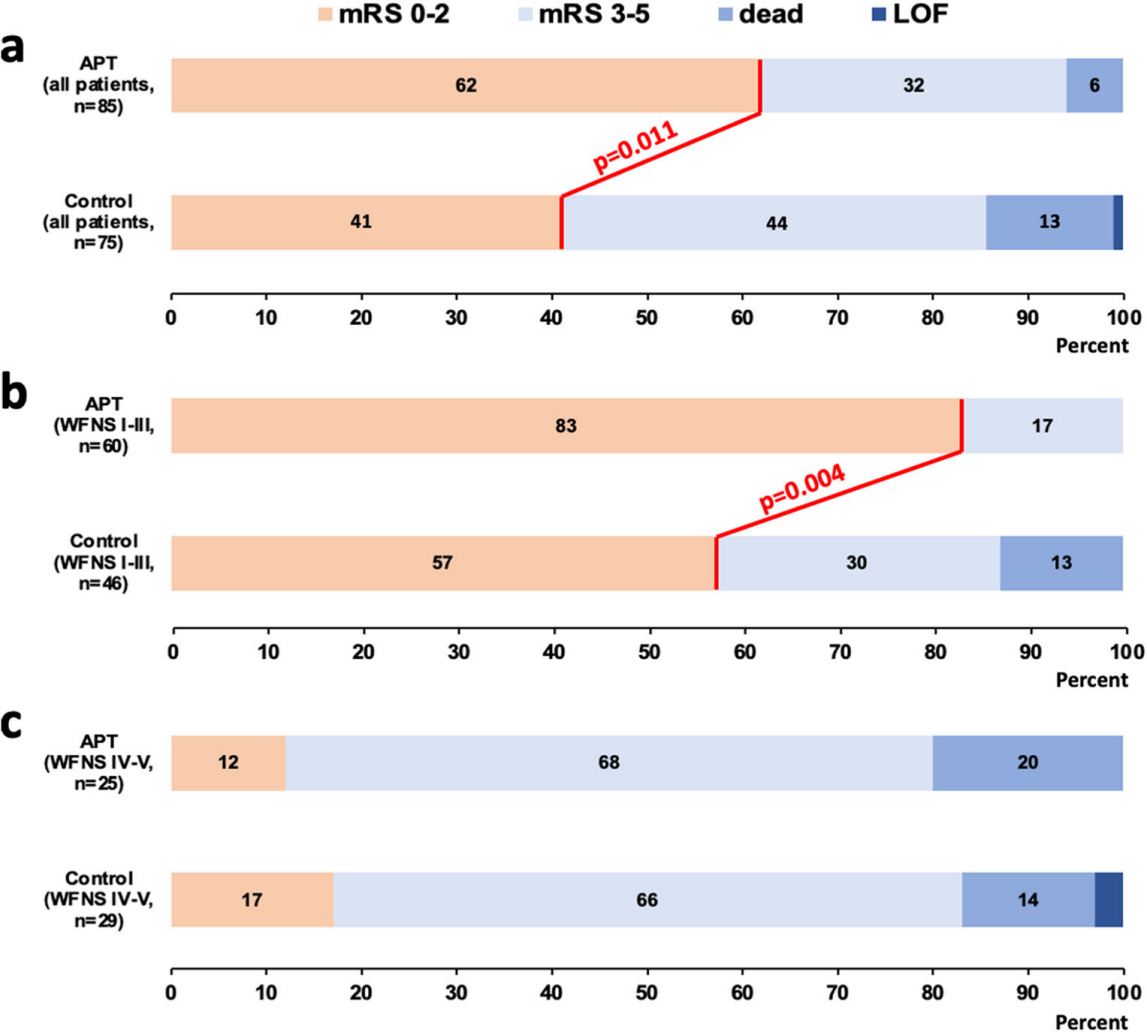
Stacked bar charts of overall functional outcome after 3 months in the control and the APT group (**a**) as well as in WFNS grade I–III (**b**) and WFNS IV–V (**c**) patients compared between the control and the APT group. The bars indicate the proportions of patients with favorable (mRS 0–2) and unfavorable outcome (mRS 3–6) with further subdivision into death (mRS 6) and loss of follow-up (LOF)

Functional outcome after 3 months is shown in Fig. 2. The rate of favorable (mRS ≤ 2) outcome (62% in the APT group vs. 41% in the control group, *P* = 0.011; Fig. 2a) was significantly higher in the APT group than in controls. Comparing the outcome of patients who were admitted with WFNS grade I–III aSAH, functional outcome was significantly better in the APT group (favorable outcome in 83% in the APT vs. 57% in the control group, *P* = 0.004: Fig. 2b), while only a small proportion of poor-grade patients (WFNS grade IV–V) in both groups showed favorable functional outcome after 3 months (12% in the APT vs. 17% in the control group, *P* = 0.708: Fig. 2c).

After adjustment for potential confounders, multivariable analysis confirmed an independent association between APT and a lower rate of unfavorable functional outcome after 3 months (*P* = 0.021, OR = 0.40, 95% CI 0.19–0.87; Table 4). In contrast, the factors “older age (> 55 years)” (*P* = 0.014, OR = 2.67, 95% CI 1.22–5.86) and “poor WFNS grade (IV–V)” (*P* < 0.001, OR = 16.23, 95% CI 6.49–40.58) increased the risk for an unfavorable functional outcome (Table 4).

**Table 4:** Logistic regression analysis of factors associated with unfavorable functional outcome (mRS ≥ 3)

	Univariable analysis		Multivariable analysis	
Parameter	OR [CI 0.95]	*P* value	OR [CI 0.95]	*P* value
Older age (> 55 years)	*2.09 [1.11–3.93]*	*0.023*	*2.67 [1.22–5.86]*	*0.014*
Male sex	0.80 [0.40–1-61]	0.534	–	–
WFNS grade 4–5	*14.25 [6.01–33.76]*	*< 0.001*	*16.23 [6.49–40.58]*	*<0.001*
Fisher scale 3	0.66 [0.35–1.24]	0.193	–	–
Occurrence of IVH	*4.80 [2.43–9.50]*	*< 0.001*	1.99 [0.81–4.89]	0.132
Aneurysm localization posterior circulation	1.17 [0.58–2.36]	0.669	–	–
Antiplatelet therapy	*0.44 [0.23–0.82]*	*0.010*	*0.40 [0.19–0.87]*	*0.021*

Data comparisons were made using univariable and multivariable analysis. Statistically significant differences are made italics (*P* < 0.05)

*mRS* modified Rankin scale, *OR* odds ratio, *CI* confidence interval, *WFNS* World Federation of Neurosurgical Societies, *IVH* intraventricular hemorrhage

Due to the significant smaller volume of DCI-related infarction in patients with DAPT, we performed a further subgroup analysis within the treatment group. When splitting the APT group into “aspirin monotherapy” and “DAPT,” univariable subgroup analysis showed that DAPT was significantly associated with a lower rate of unfavorable outcome at 3 months (*P* = 0.021, OR = 0.43, 95% CI 0.21–0.88) while aspirin monotherapy still showed a statistical trend (*P* = 0.068, OR = 0.44, 95% CI 0.18–1.06).

## Discussion

Post-interventional APT was found to be an independent predictor of a better functional outcome after 3 months in the aSAH patients of our cohort. These results are in line with previous studies reporting on improved outcome in aSAH patients who received antiplatelets after aneurysm treatment [4, 11, 14]. Although the exact mode of action by which antiplatelets influence outcome after aSAH is not yet clarified, it has been previously suggested that platelet inhibitors reduce the risk of DCI and thereby have a beneficial effect on clinical outcome. Accordingly, several studies investigated a possible preventive effect of different antiplatelet agents on the development of DCI, but those trials are quite heterogeneous and thus difficult to interpret. A former systematic review of clinical studies, investigating microsurgically treated patients only, concluded that APT reduces the risk of DCI resulting in an increased rate of good outcome after aSAH [14]. However, only three of the five included trials provided data on the occurrence of DCI and only one of them used aspirin as trial medication. Noteworthy, this small, randomized trial found an equal distribution of clinical and radiologic DCI in both groups [20].

A more recent Cochrane review demonstrated a non-significant trend toward a lower incidence of DCI and a better functional outcome among patients treated with antiplatelets after aSAH [15]. But again, the validity of this meta-analysis is limited due to the differences of the studies included such as the trial medication used or the start of antiplatelet treatment. Moreover, the majority of these patients were treated by neurosurgical clipping, so that the results are hardly transferable to endovascularly treated aSAH patients, who constitute the group of patients that usually needs APT.

In our cohort of endovascularly treated patients, we found no significant difference with regard to the overall occurrence of clinical DCI and DCI-related infarction between patients receiving APT and those without. Likewise, the occurrence of angiographic CVS was comparable in both groups. These results seem surprising at first, especially in view of the better functional outcome of the APT group in our study. But there are several indications in the literature that improved outcome through antiplatelets cannot be equated with the prevention of DCI. A randomized controlled trial assessing whether aspirin administration reduces the risk of DCI in SAH patients failed to demonstrate such an effect and concluded that aspirin does not reduce the occurrence of DCI. Still, functional outcome tended to be better in patients who received aspirin, especially after endovascular aneurysm treatment [45]. Similarly, the largest and most recent meta-analysis to date analyzing the use of antiplatelet drugs among 2822 patients with aSAH found significantly reduced rates of poor outcome and mortality among the APT group, but antiplatelet use did not significantly decrease DCI or CVS rates [4]. In this study, the majority of patients (75%) were treated endovascularly and 95% of the patients received aspirin as post-interventional APT. Interestingly, this meta-analysis found a trend toward lower DCI among the subgroup of endovascularly treated patients. Similar positive effects of APT on the occurrence DCI were reported by two recent series of endovascularly treated aSAH patients [11, 29]. The results of our study, however, do not confirm these findings in endovascularly treated aSAH patients as we could not demonstrate any differences in the overall occurrence of DCI and angiographic CVS in our collective. There are indications in the literature that the reduction of DCI after antiplatelet administration tended to be lower in patients with a high Fisher score (Fisher 3–4) [4]. As the amount of cisternal blood represents an independent risk factor for CVS and DCI [6, 7, 27], it seems reasonable that the various pathophysiological mechanisms that contribute to the development of CVS and DCI might be enhanced by the severity of the initial bleeding event. In this case, the beneficial effects of antiplatelets might be less pronounced or the chosen dosage might be too low. As the vast majority of patients in our cohort presented with Fisher grade 3–4, this condition might have influenced our results.

Due to the discrepancy between improved outcome and absence of overall CVS and DCI reduction in patients with post-interventional APT, we performed a detailed analysis of the severity and the distribution patterns of angiographic CVS and of DCI-related infarction. Interestingly, we found no significant differences between the groups with regard to the severity, the vessels or vascular territories affected, or any other investigated parameter. The analysis of the infarct lesion volumes demonstrated a non-significant statistical trend toward a higher lesion volume in the control group, and the descriptive lesion analyses in our study indicated that the patients in the control group developed more often larger infarctions with a predominance in the left hemisphere. Moreover, the results of the subgroup analysis suggest that especially the DAPT treatment might have a protective effect. The beneficial effect of APT on functional outcome in our cohort might therefore be mediated by the reduction of the lesion size, especially when DAPT was administered which was the case in the majority (2/3) of our patients receiving antiplatelet medication. In this context, it is interesting that PCT examinations in the APT group demonstrated DCI-related cerebral hypoperfusion significantly less often compared with the control group.

Previous studies proposed that the positive impact of APT on clinical outcome might be associated with a decreased occurrence of microthrombi [4, 45]. Several clinical and autopsy studies reported that the formation of microthrombi and the associated impairment of cerebral microcirculation contributes to the development of DCI and cerebral infarction after SAH [34, 41, 42, 49]. An autopsy study showed that the amount of microemboli is significantly increased in SAH patients with large areas of DCI-related cerebral ischemia [42]. APT might therefore reduce the formation or the amount of microemboli and thus the extension of DCI-related cerebral infarction in aSAH patients. While aspirin irreversibly inhibits the enzyme cyclooxygenase (COX) and thus the subsequent biosynthesis of prostaglandins and thromboxane, clopidogrel blocks platelet aggregation through the P2Y_12_-receptor pathway [16]. These mechanisms lead to synergistic effects with regard to the inhibition of platelet aggregation. The combination of the two drugs has been shown to be more effective than aspirin alone in reducing the risk of ischemic events in patients with acute coronary syndromes [2] as well as the risk of recurrent stroke in the acute phase after an ischemic event in neurological patients [23, 32]. Our study provides indications that such an additive beneficial effect from DAPT could also apply for DCI-related infarctions in aSAH patients.

So far, data about potential benefits and risks associated with DAPT after aSAH are scarce and inconsistent. A recent study of patients presenting with Hunt and Hess grade I-III showed a significantly reduced risk for clinical CVS and DCI but could not demonstrate a positive effect of DAPT on functional outcome [29]. A second retrospective study of 329 aSAH patients receiving either aspirin (92.4%) or DAPT (7.4%) demonstrated decreased DCI rates and improved functional outcome in the entire APT group compared with the control group. However, a subgroup analysis of the DAPT cohort showed no significant impact on the occurrence of DCI. Moreover, patients with DAPT had higher in-hospital mortality, lower favorable outcome, and an increased risk of major bleeding events [11]. A second study, comparing the outcomes among aSAH patients who received aspirin monotherapy vs. DAPT, also failed to demonstrate a superior beneficial effect of DAPT reporting a trend toward higher bleeding complication rates in the DAPT group as well [11, 51].

In view of these findings, the potential risks of DAPT in neurosurgical patients must be discussed. Several studies show increased rates of intracranial bleeding complications due to single-antiplatelet use in aSAH patients [14, 15, 44]. With regard to DAPT, two recent studies by *Hudson* et al. demonstrate a higher risk for radiographic hemorrhage associated with external ventricular drain or VP shunt placement in aSAH patients requiring DAPT due to stent-assisted coiling or flow diversion. However, the risk of clinically significant bleeding complications was considered low [21, 22]. A recent meta-analysis [5] reported that ventriculostomy-related bleeding rates were significantly higher among patients receiving APT and that the rate of major hemorrhage was higher after DAPT compared with single APT. But the overall rate of major hemorrhage was also low in this study. In our study, we found no significant differences in the rate of hemorrhagic complications between the investigated groups. Major bleeding events occurred only in a small number of patients. However, all of those individuals received DAPT, and the size of our study groups was probably too small to detect statistically significant differences of major bleeding events in patients with DAPT. Our results in general confirm the assumption that clinically relevant bleeding events are rare in aSAH patients with APT, but are tending to occur more often in DAPT than in mono APT.

Our results confirm that a poor WFNS grade (IV–V) is a strong predictor of DCI-related infarction and unfavorable outcome after aSAH. Interestingly, our clinical outcome analysis revealed that only APT patients presenting with WFNS grade I–III showed a better functional outcome, whereas the outcome of patients with WFNS grade IV–V was similar between both groups. Our results suggest that post-interventional APT fails to improve functional outcome in WFNS grade IV–V patients, at least in our study cohort. Poor-grade aSAH patients are not only characterized by a particularly severe early brain injury (EBI), but also by the risk of developing severe medical complications during the acute clinical course. The extent of platelet activation and inflammation has been shown to be related to the severity of EBI after aSAH [19]. One could therefore speculate that the antiplatelet effect or the mode of action of the drugs used was insufficient in these severely affected patients or that the dosage might not have been high enough. In this context, a major limitation of our study must be discussed. The individual responder status to APT has not been routinely analyzed especially not in patients receiving aspirin monotherapy. This issue is at the same time a major shortcoming of previous studies dealing with APT after aSAH as even randomized and prospective studies have not acknowledged APT responder status [15, 45]. It is well known that the individual responsiveness to antiplatelet drugs varies among patients and is influenced by genetic polymorphism but also by clinical factors such as platelet hyperactivity, drug-drug interactions, and poor adsorption [1, 43]. The reported rates of aspirin and clopidogrel range widely in the literature [8], but it has been shown that impaired responsiveness to aspirin is common in acute brain ischemia and is also associated with worse neurological deficits at stroke onset, early clinical deterioration, and poorer functional outcome [40]. In analogy to these findings, impaired antiplatelet responsiveness might also be associated with the severity of EBI in poor-grade SAH patients suggesting that there might have been an overestimation of APT-responders in our study. Given the possibility that an early inhibition of platelet function during EBI might be crucial with regard to the further course of the disease, poor WFNS grade patients in particular might benefit from a higher aspirin dosage or the additional administration of clopidogrel. In this context, DAPT with an early loading dose of aspirin and clopidogrel might be beneficial, and our study confirmed a sufficient response to both drugs in the majority of patients. Future prospective studies should acknowledge this important aspect.

Our study is moreover limited by its retrospective design and the small cohort size affecting the clinical significance of our results. As we excluded patients who received surgical aneurysm treatment, our results are not transferable to all aSAH patients. However, patients who underwent aneurysm clipping are not well suited as a control group, as craniotomy, cisternal blood removal, or microsurgical manipulation of the brain and its vasculature might have an incalculable impact on the study results. As the investigated groups in our study were defined according to the need of post-interventional APT, another limitation is given by a certain selection bias with regard to the type of endovascular aneurysm treatment as a relevant number of patients in the APT group received stent-assisted coiling. The decision on what type of endovascular therapy was used might have been influenced not only by the complexity of the aneurysm configuration but also by the clinical factors or the individual assessment of the interventional neuroradiologist. The use of stent-assisted coiling may carry some beneficial effects regarding the functional outcome in the APT group, as patients who received coiling only without post-interventional APT might be at higher risk for ischemic cerebral events.

Compared with previous studies, we used DCI-related infarction as an independent outcome measure in multivariable analysis instead of overall clinical and/or radiological DCI. Due to the heterogeneity of the study cohort consisting of awake and analgosedated aSAH patients, data on clinical DCI were only partially available limiting the power of statistical analysis. However, we assume that the occurrence of DCI-related cerebral infarction has a greater impact on functional outcome than temporary neurological deterioration associated with DIND. Moreover, a strict clinical definition of DCI in previous studies often resulted in an underrepresentation of poor-grade SAH patients, who at the same time are at the highest risk for ischemic complications [39].

Finally, reliable data on previous antiplatelet long-term medication or one-time antiplatelet intake in connection with the acute headache event were not available for all patients and therefore could not be considered in the analysis. Thus, previous antiplatelet medication before admission might also have influenced the results of this study.

## Conclusion

Post-interventional APT was associated with a lower rate of unfavorable outcome in our aSAH cohort without an increased risk for intracranial bleeding events. The overall incidence of angiographic CVS or DCI-related infarction was not reduced in the APT group. However, with regard to the volume of DCI-related infarctions, our results suggest that APT, especially DAPT, may reduce the lesion’s size and thereby improves clinical outcome. Our results underline the need for prospective randomized trials to assess and compare potential benefits and risks of mono- and DAPT in patients with aSAH and to answer the clinical question of whether the use of APT is indicated in patients after aSAH in order to improve clinical outcome.

## Data Availability

The data that support the findings of this study are available from the corresponding author, upon reasonable request.
